# NMR Metabolomics in Veterinary Medicine: From Biomarker Discovery to Clinical Implementation

**DOI:** 10.3390/vetsci13090921

**Published:** 2026-09-07

**Authors:** Alvaro Pérez-Collar, Carlos Velasco, Javier Bezos, Jose Luis Izquierdo-Garcia

**Affiliations:** 1 Grupo de RMN e Imagen en Biomedicina, Instituto Pluridisciplinar, Universidad Complutense de Madrid, 28040 Madrid, Spain; alvarp16@ucm.es; 2 Centro de Vigilancia Sanitaria Veterinaria (VISAVET), Universidad Complutense de Madrid, 28040 Madrid, Spain; carvelas@ucm.es (C.V.); jbezosga@ucm.es (J.B.); 3 Departamento de Sanidad Animal, Facultad de Veterinaria, Universidad Complutense de Madrid, 28040 Madrid, Spain; 4 Departamento de Química en Ciencias Farmacéuticas, Facultad de Farmacia, Universidad Complutense de Madrid, 28040 Madrid, Spain; 5 CIBER de Enfermedades Respiratorias (CIBERES), Instituto de Salud Carlos III, 28029 Madrid, Spain

**Keywords:** NMR metabolomics, veterinary medicine, biomarkers

## Abstract

Nuclear magnetic resonance (NMR)-based metabolomics has emerged as a robust and reproducible analytical platform for investigating the molecular mechanisms underlying disease and identifying candidate biomarkers with potential clinical relevance. Although initially developed for biomedical research, its application in veterinary medicine has expanded rapidly during the last decade, driven by the growing demand for precision medicine approaches capable of improving disease diagnosis, prognosis and therapeutic monitoring. This review summarizes the current landscape of NMR metabolomics in veterinary medicine, highlighting its applications across major clinical areas, including oncology, infectious, respiratory, digestive, renal, endocrine and musculoskeletal diseases. Collectively, the available evidence demonstrates that NMR metabolomics consistently identifies disease-associated metabolic alterations, providing novel insights into pathophysiology while supporting biomarker discovery in naturally occurring animal diseases. Beyond current clinical applications, we discuss the major challenges that continue to limit routine implementation, including biological variability, standardization of analytical workflows, multicentre validation and clinical translation. Particular attention is given to recent technological advances, such as benchtop NMR instrumentation, artificial intelligence-assisted data analysis, multi-omics integration and the development of collaborative networks and standardized platforms. Together, these advances position NMR metabolomics as a promising technology to support precision veterinary medicine, facilitating its transition from biomarker discovery to routine clinical implementation.

## Simple Summary

Animal diseases can be difficult to detect early, and many current tests provide limited information about how a disease is affecting the whole body. This review examines whether nuclear magnetic resonance, a laboratory method that measures many small chemicals in blood, urine, milk and other samples, could improve disease detection and monitoring in veterinary medicine. Published studies have found chemical patterns associated with cancer, infections, respiratory and digestive disorders, kidney disease, hormonal conditions and joint disease in several animal species. However, most studies involved relatively small groups of animals, and their findings have rarely been confirmed in separate populations. Smaller and easier-to-use instruments, together with computer-assisted analysis, may make this approach more accessible, but routine use will require larger studies conducted at several centres, standard procedures, comparison with existing tests and evidence that it is affordable and improves veterinary decisions. If these requirements are met, this technology could support earlier diagnosis, better treatment selection, reduced unnecessary antimicrobial use, improved animal welfare and lower economic losses in livestock production.

## 1. Introduction

Veterinary medicine is undergoing a profound transformation driven by the growing recognition that animal, human and environmental health are intrinsically interconnected within the One Health framework. Beyond safeguarding animal health and welfare, modern veterinary medicine plays a central role in controlling zoonotic diseases, ensuring food safety, combating antimicrobial resistance and supporting sustainable livestock production. As interactions between humans, animals and the environment become increasingly complex, conventional diagnostic approaches based primarily on clinical examination and routine laboratory testing are no longer sufficient to address many of the current challenges. Consequently, veterinary medicine is progressively embracing the principles of precision medicine, supported by advances in molecular sciences, analytical technologies and digital health that provide increasingly objective and comprehensive information for disease prevention, diagnosis, prognosis and therapeutic decision-making [1,2].

Precision veterinary medicine seeks to characterize disease at the molecular level by integrating biomarkers with clinical, imaging and environmental information to support individualized patient management. This approach is particularly attractive because companion animals naturally develop many diseases that closely resemble their human counterparts, strengthening the role of comparative medicine, while livestock production increasingly demands objective tools capable of improving animal health, welfare and production efficiency. At the same time, veterinary medicine presents unique challenges arising from the remarkable diversity among species, breeds, production systems and environmental exposures, making analytical robustness and reproducibility essential prerequisites for successful clinical implementation. These requirements have accelerated the adoption of high-throughput omics technologies capable of characterizing biological systems at multiple molecular levels and identifying disease-associated molecular signatures with unprecedented depth, establishing the foundations for precision veterinary medicine [3,4,5].

The successful implementation of precision veterinary medicine depends on the availability of robust molecular biomarkers capable of objectively characterizing disease throughout its natural history. Ideally, these biomarkers should enable early diagnosis, improve disease classification, predict prognosis, monitor therapeutic response and ultimately support evidence-based clinical decision-making [6]. Despite significant advances in veterinary diagnostics, however, many diseases continue to rely on conventional clinical assessment and routine laboratory testing, which frequently lack sufficient sensitivity and specificity, particularly during the early stages of disease [7]. Consequently, there is growing interest in molecular profiling approaches capable of improving diagnostic accuracy while simultaneously providing deeper insight into the biological mechanisms underlying disease development and progression [5,8].

This unmet need spans virtually every area of veterinary medicine. In oncology, molecular biomarkers are required to support early diagnosis, patient stratification and treatment selection. In infectious diseases, they can facilitate the differentiation between pathogen exposure, latent infection and active disease while enabling treatment monitoring. Likewise, chronic disorders, including kidney, endocrine and inflammatory diseases, would benefit from biomarkers capable of detecting disease before irreversible tissue damage occurs and objectively monitoring disease progression over time. Across these diverse clinical scenarios, the common challenge is the identification of biomarkers that reflect the underlying biological processes driving disease, rather than simply measuring structural damage or isolated biochemical abnormalities [7,9].

The development of high-throughput omics technologies has fundamentally transformed biomarker discovery by enabling the comprehensive characterization of biological systems at multiple molecular levels. Genomics, transcriptomics and proteomics have greatly advanced our understanding of disease mechanisms by identifying molecular alterations associated with genetic susceptibility, gene regulation and protein function. Although these approaches provide invaluable insights into the molecular basis of disease, they primarily characterize genetic and regulatory potential rather than the organism’s dynamic functional phenotype [10,11]. Initial investigations established the feasibility. In contrast, metabolomics investigates the small molecules involved in cellular metabolism, providing a context-dependent biochemical representation of phenotype shaped by genetic background, nutritional and physiological status, environmental exposures, microbiome composition and disease processes. As a molecular layer closely linked to phenotype, metabolomics has become an important component of precision medicine, offering opportunities for biomarker discovery, disease stratification and clinical translation [12].

Throughout this review, the term “metabolic signature” refers to a multivariate pattern of metabolites associated with a biological or clinical state. An individual metabolite or metabolic signature associated with a condition in an exploratory study is referred to as a “candidate biomarker”. The term “validated biomarker” is reserved for markers whose analytical performance and clinical validity have been confirmed in independent, clinically representative populations.

Among the analytical platforms used in metabolomics, nuclear magnetic resonance (NMR) spectroscopy stands in a unique position because of its analytical robustness, quantitative performance and exceptional interlaboratory reproducibility [13]. While mass spectrometry provides greater analytical sensitivity and broader metabolite coverage, NMR offers highly reproducible and inherently quantitative measurements with minimal sample preparation, excellent instrument-to-instrument comparability and remarkable long-term analytical stability [14,15] (Table 1). These characteristics have enabled the establishment of standardized analytical workflows, multicenter studies and standardized NMR platforms used in clinical research, making NMR particularly well suited for longitudinal investigations and future routine clinical implementation [13].

Over the past decade, veterinary NMR metabolomics has evolved from exploratory proof-of-concept studies into an increasingly mature discipline with growing clinical relevance across multiple areas of veterinary medicine [16]. This evolution, together with recent advances in benchtop NMR technology, is creating new opportunities to extend metabolomic analyses beyond specialized research laboratories and facilitate their integration into routine veterinary practice [17].

This review provides a comprehensive overview of the current landscape of veterinary NMR metabolomics, emphasizing its evolution from biomarker discovery to clinical implementation. We first summarize the principal clinical applications across major veterinary diseases before discussing the analytical, biological and translational challenges that currently limit routine clinical adoption. Finally, we examine emerging technologies and collaborative infrastructures that are expected to accelerate the implementation of NMR metabolomics within precision veterinary medicine.

## 2. Review Methodology

During the preparation of this manuscript, the authors used ChatGPT (OpenAI, GPT-5.5) for the purposes of language editing, improving clarity and readability and generating conceptual ideas for figures and graphical abstracts. The authors have reviewed and edited the output and take full responsibility for the content of this publication.

### 2.1. Literature Search Strategy

This article was designed as a narrative review rather than as a systematic or scoping review. A structured literature search was conducted in PubMed, Scopus and Web of Science for publications appearing between January 2006 and 31 May 2026. The search combined the terms (“nuclear magnetic resonance” OR “NMR” OR “benchtop NMR” OR “low-field NMR”) AND (“metabolomics” OR “metabonomics”) with general veterinary and species-specific keywords, including “veterinary”, “biomarker”, “animal disease”, “dog”, “canine”, “cat”, “feline”, “horse”, “equine”, “cattle”, “bovine”, “sheep”, “ovine”, “goat”, “caprine”, “pig” and “swine”. Additional searches combined these terms with the principal clinical areas addressed in this review, including oncology, infectious disease, mastitis, sepsis, tuberculosis, paratuberculosis, respiratory disease, gastrointestinal disease, kidney disease, endocrine disease and musculoskeletal disease. The reference lists of relevant original articles and reviews were also manually screened to identify additional publications.

### 2.2. Eligibility Criteria and Evidence Synthesis

Peer-reviewed, English-language articles investigating NMR-based metabolomics in naturally occurring diseases or clinically relevant conditions affecting veterinary species were considered eligible for inclusion. Original veterinary NMR studies constituted the core clinical evidence. Reviews, methodological publications, clinical guidelines and selected human studies were included when they provided relevant analytical, biological or translational context.

Retrieved records were screened for relevance based on title and abstract, followed by full-text assessment of potentially eligible studies. Duplicate records identified across databases were removed before screening.

Studies focused exclusively on food authentication, food composition or production characteristics without a direct animal-health application were excluded, as were purely experimental animal models lacking clear veterinary relevance. Studies based exclusively on analytical platforms other than NMR were excluded from the core evidence synthesis but were considered when required for comparison or biological interpretation.

For each eligible study, relevant information was extracted regarding veterinary species, clinical condition, study population and sample size, biological matrix, NMR platform and acquisition strategy, metabolomics workflow, spectral preprocessing and statistical methods, metabolites or metabolic pathways identified, and the principal diagnostic, prognostic or biological findings. Attention was given to the identification of biomarkers and to the reported diagnostic performance of metabolomic signatures, when available.

The evidence was organized according to the main areas of veterinary clinical practice and synthesized narratively, with emphasis on disease-associated metabolic alterations, candidate biomarkers, biological interpretation, diagnostic or prognostic potential, and the translational applicability of NMR-based metabolomics. Given the heterogeneity among studies in terms of species, diseases, biological matrices, NMR methodologies, study designs and statistical approaches, a quantitative meta-analysis was not considered.

## 3. Clinical Applications of NMR Metabolomics

The application of NMR metabolomics has expanded rapidly across veterinary medicine, generating a growing body of evidence in numerous naturally occurring diseases affecting both companion and production animals. Although many studies remain exploratory and are frequently limited by relatively small cohorts, they consistently demonstrate that disease-associated metabolic fingerprints can be identified across a wide range of pathological conditions. Beyond biomarker discovery, these investigations have provided valuable insights into disease mechanisms, patient stratification, prognosis and therapeutic monitoring [16,18].

To facilitate discussion, the available evidence is organized according to the major areas of veterinary clinical practice (Table 2). Studies directly applying NMR metabolomics to veterinary species constitute the primary evidence base, whereas findings from human studies and complementary analytical or omics platforms are included only as biological, mechanistic or translational context and are explicitly identified as such. Although direct veterinary NMR studies show that disease-associated metabolic alterations can be detected across different species and biological matrices, the evidence remains heterogeneous and predominantly exploratory.

Most studies were conducted at a single centre using small or moderately sized cohorts, and independent external validation was uncommon. Species, breed, age, sex, diet, physiological status, treatment, comorbidities and sample-collection conditions represent important potential confounders. Moreover, experimentally induced disease may not fully reflect naturally occurring cases, while diagnostic performance is generally estimated through internal model evaluation and may be optimistic in high-dimensional, small-cohort studies. Accordingly, most reported metabolic alterations should be regarded as candidate biomarkers or metabolic signatures until confirmed using standardized protocols and locked models in independent, preferably multicentre cohorts.

### 3.1. NMR Metabolomics in Veterinary Oncology

Cancer represents one of the leading causes of morbidity and mortality in companion animals and has become an increasingly important challenge for both veterinary medicine and comparative oncology. Similarly to humans, dogs and cats spontaneously develop a broad spectrum of neoplasms within an intact immune system while sharing environmental exposures, disease progression and many biological characteristics with their human counterparts, making them valuable spontaneous models for comparative oncology [19]. As a result, canine cancer, including mammary tumors, lymphoma, osteosarcoma, melanoma, urothelial carcinoma and hemangiosarcoma, have become increasingly important for investigating tumor biology, biomarker discovery and the development of novel therapeutic strategies. Many of these tumors closely resemble human malignancies with respect to histopathological features, metastatic behavior, molecular heterogeneity and the activation of conserved oncogenic pathways, further reinforcing their translational relevance [19,20].

Before considering the evidence generated directly using NMR in veterinary species, findings from genomic, transcriptomic, proteomic and mass spectrometry-based studies are briefly summarized to provide biological and mechanistic context.

The biological basis for applying metabolomics to veterinary oncology is further supported by evidence from other omics disciplines. Large-scale genomic, transcriptomic and proteomic studies have demonstrated that canine tumors share many of the molecular alterations described in human cancer. For example, multi-omics analyses of canine mammary tumors have identified recurrent dysregulation of PI3K–Akt signaling, p53-associated pathways, immune microenvironment remodeling and transcriptional programs that closely parallel those observed in human breast cancer [21,22]. Likewise, transcriptomic and proteomic studies in canine osteosarcoma and genomic analyses of hemangiosarcoma have revealed alterations involving cellular bioenergetics, oxidative stress, angiogenesis, inflammation and PI3K/RAS signaling, including recurrent abnormalities affecting PIK3CA, NRAS, TP53 and PTEN [23,24,25,26]. Importantly, many of these molecular alterations converge on common metabolic processes—including glucose utilization, amino acid metabolism, lipid biosynthesis, mitochondrial function and redox homeostasis—providing a strong biological rationale for metabolomic investigations aimed at characterizing the functional phenotype of veterinary cancers.

The metabolic consequences of these molecular alterations have been extensively investigated using mass spectrometry-based metabolomics, providing further evidence that metabolic reprogramming is a hallmark of spontaneous canine cancers. Across multiple tumor types, including mammary tumors, lymphoma, melanoma and urothelial carcinoma, metabolomic studies have consistently identified alterations affecting central carbon metabolism, glucose utilization, amino acid turnover, lipid remodeling and mitochondrial function, closely paralleling the metabolic hallmarks described in human oncology [27,28,29,30]. Furthermore, Gas chromatography–mass spectrometry (GC–MS) profiling of sphere-forming cells derived from canine mammary adenocarcinoma identified distinct metabolic signatures associated with stem-like tumor cell populations, highlighting the close relationship between metabolic adaptation, cellular plasticity and tumor aggressiveness [31]. Together, these studies provide compelling evidence that metabolic dysregulation represents a fundamental feature of naturally occurring canine cancer, thereby establishing a strong biological foundation for NMR-based metabolomic investigations.

Direct evidence specifically generated using NMR metabolomics in veterinary oncology is more limited and is derived predominantly from exploratory studies in dogs. Nevertheless, these studies demonstrate the feasibility of detecting tumour-associated metabolic alterations in accessible biofluids, although they do not yet establish reproducibility or diagnostic performance in independent external cohorts. The first NMR metabolomics study in this field identified differences in urinary metabolic profiles between dogs with urothelial carcinoma and healthy controls, including perturbations related to energy metabolism, amino acid utilization and membrane-associated metabolites [32]. This study demonstrated the feasibility of using NMR to characterize tumour-associated metabolic alterations in urine but did not establish diagnostic performance in an independent cohort.

A subsequent study generated a publicly available urinary NMR metabolomics dataset comprising 156 female dogs, including 20 dogs with benign mammary tumours, 87 with malignant tumours and 49 healthy controls [33]. The study annotated 55 urinary metabolites and reported group-associated alterations involving amino acid metabolism, energy production and lipid metabolism. This dataset represents a valuable resource for methodological development and future validation studies. However, it was not designed as an independent validation of a predefined diagnostic model, and differences in age and breed composition between groups should be considered when interpreting the reported metabolic alterations.

Subsequently, serum NMR metabolomics was successfully applied to dogs with multicentric lymphoma, further extending the clinical applicability of NMR beyond epithelial tumors [34]. Compared with healthy controls (*n* = 25), affected dogs (*n* = 37) exhibited a distinct metabolic phenotype characterized by alterations in glucose metabolism, lactate production, amino acid turnover and lipid-related metabolites, including differences in lactate, glucose, N-acetyl glycoproteins, scyllo-inositol and choline-containing compounds. Notably, many of these metabolic alterations closely resemble those described in human hematological malignancies [35], further reinforcing the value of spontaneous canine lymphoma as a comparative oncology model and highlighting the translational relevance of veterinary NMR metabolomics.

Across these studies, partially overlapping alterations in energy metabolism, amino acid turnover and lipid metabolism have been reported in different canine tumour types and biological matrices. These pathways are also consistent with established features of cancer metabolism [36], although similarity to findings from human or non-NMR studies should be interpreted as biological support rather than independent validation. Given the small number of veterinary NMR studies, differences in study design and the absence of external validation cohorts, these metabolic patterns cannot yet be regarded as reproducible diagnostic signatures.

More recently, the application of NMR metabolomics has begun to extend beyond biomarker discovery towards clinically oriented diagnostic strategies. Rather than focusing exclusively on individual metabolites, recent studies have demonstrated the potential of integrating complete NMR-derived metabolic fingerprints with machine learning algorithms for automated disease classification. In a cohort of 139 client-owned dogs—24 healthy animals and 115 dogs affected by different conditions, including 34 cancer cases—the integration of NMR-derived serum metabolite and lipoprotein measurements with haematological and clinical variables enabled discrimination between healthy and diseased animals using repeated internal cross-validation [37]. These results support the feasibility of machine-learning-assisted disease screening, although the reported performance remains to be confirmed in an independent external cohort.

Although veterinary NMR oncology remains an emerging field, the progression observed over the past decade illustrates a clear evolution from exploratory proof-of-concept studies towards increasingly standardized and clinically relevant applications. Initial investigations established the feasibility of detecting tumour-associated metabolic alterations in accessible biofluids, whereas more recent studies have expanded publicly available datasets, reported related disease-associated metabolic alterations across different tumour types and incorporated computational approaches for multivariate classification. However, these developments do not constitute independent external validation of the reported metabolic signatures or diagnostic models. Collectively, these advances demonstrate that veterinary NMR metabolomics is progressively evolving from a predominantly exploratory research tool into a clinically oriented technology with the potential to support cancer diagnosis, patient stratification and precision oncology in companion animals.

### 3.2. NMR Metabolomics in Infectious Diseases

Infectious diseases remain among the leading causes of morbidity, mortality and economic loss in both companion animals and livestock, while also representing a major challenge within the One Health framework through the emergence of zoonotic pathogens and antimicrobial resistance. Despite considerable advances in microbiological diagnostics and molecular detection techniques, traditional approaches frequently provide limited information regarding disease severity, host response against the infection and treatment efficacy. In this context, metabolomics has emerged as a complementary methodology capable of capturing the biochemical consequences of infection by simultaneously monitoring coordinated alterations across multiple metabolic pathways, rather than focusing exclusively on pathogen detection [38,39].

The close relationship between cellular metabolism and immune function has led to the emergence of the field of immunometabolism, which investigates how metabolic pathways regulate immune cell activation, differentiation and effector function [40]. Activation of both innate and adaptive immune responses is accompanied by profound metabolic adaptations that sustain cytokine production, antimicrobial activity, immune cell proliferation and tissue repair. Consequently, infectious diseases induce coordinated metabolic alterations that reflect not only pathogen-specific mechanisms but, more importantly, the integrated physiological response of the host to infection [41,42].

Metabolomics studies in human infectious diseases have consistently shown that diverse pathogens induce perturbations in a relatively limited number of interconnected metabolic pathways, including glycolysis, mitochondrial energy metabolism, amino acid utilization, lipid metabolism and oxidative stress [43,44]. In addition to these conserved metabolic adaptations, alterations in host–microbiota co-metabolism and inflammatory mediators have been recognized as important determinants of disease progression and clinical outcome [45]. Collectively, these studies demonstrate that metabolomic profiling primarily reflects the integrated physiological response of the host to infection, rather than the direct metabolic activity of the invading pathogen, providing a systems-level perspective on infectious disease pathophysiology. The following sections illustrate how these principles have been applied to the study of the major infectious diseases investigated in veterinary medicine, highlighting both disease-specific metabolic signatures and the common metabolic adaptations associated with host responses to infection.

#### 3.2.1. Mastitis

Mastitis is one of the most economically important diseases affecting dairy production worldwide, causing substantial economic losses through reduced milk yield, impaired milk quality, compromised animal welfare and increased antimicrobial use [46]. This multifactorial disease is caused by a wide range of bacterial, fungal and, less frequently, viral pathogens, while host factors, management practices and hygiene conditions also play major roles in disease development [47]. Conventional diagnosis relies primarily on bacteriological culture, molecular detection techniques and somatic cell counts. However, these approaches present important limitations. Potential pathogens may also be detected in healthy mammary glands without causing clinical disease, whereas somatic cell counts are influenced by physiological and environmental factors, including stage of lactation, parity and herd management, reducing their specificity for diagnosing both clinical and subclinical mastitis [48,49]. Consequently, there is increasing interest in complementary biomarkers aimed at objectively characterizing disease activity, monitoring progression and evaluating therapeutic response.

Metabolomics has emerged as a valuable systems biology approach for investigating the complex biochemical response associated with mastitis [46,47]. Rather than focusing exclusively on pathogen identification, metabolomic profiling characterizes the integrated metabolic response of mammary tissue and the host immune system during infection. Human studies have demonstrated that mastitis is consistently associated with alterations in energy metabolism, amino acid utilization, lipid metabolism and inflammatory pathways, reflecting coordinated metabolic adaptations that accompany immune activation [44,45]. Similar metabolic alterations have subsequently been reported in veterinary species. In dairy cattle, milk NMR metabolomics has consistently identified perturbations involving carbohydrate metabolism, energy production, amino acid turnover and membrane metabolism during both clinical and subclinical mastitis, demonstrating the ability of NMR spectroscopy to detect disease-associated metabolic fingerprints directly in milk [50,51]. In addition, several metabolites, including lactate, citrate, acetate and specific amino acids, have emerged as potential biomarkers of mammary inflammation and gland dysfunction, highlighting the diagnostic potential of NMR metabolomics in one of the most clinically relevant diseases affecting dairy production.

Beyond biomarker discovery, NMR metabolomics has also contributed to a better understanding of mastitis pathophysiology. Longitudinal studies have shown that the metabolic response evolves throughout disease progression and gradually returns towards physiological conditions following successful treatment, highlighting the potential of NMR metabolomics for monitoring therapeutic response and disease resolution [51]. Furthermore, although pathogen-specific metabolic signatures have been reported, many of the observed alterations reflect conserved inflammatory and immunometabolic responses of the host, reinforcing the concept that metabolomic profiling primarily characterizes host physiology rather than the infecting microorganism itself.

More recently, the integration of NMR metabolomics with complementary approaches, including microbiome analyses and other omics technologies, has provided a more comprehensive understanding of mastitis biology. These integrative studies suggest that combining metabolic, microbial and immunological information may improve disease stratification and facilitate the identification of more robust candidate biomarkers for diagnosis and prognosis [52].

Among veterinary infectious diseases, mastitis represents a comparatively well-studied application of NMR metabolomics. Several independent studies have reported partially overlapping alterations in milk metabolites related to energy metabolism, amino acid turnover and mammary inflammation [50,51]. Together with the availability of milk as a readily accessible and non-invasive matrix/type of sample, these findings support the potential use of NMR for longitudinal metabolic monitoring. However, the studies differ in pathogen distribution, clinical definitions, stage of lactation, breed, production system, disease severity and analytical methodology. Consequently, the apparent concordance of the reported alterations should not be interpreted as evidence of validated diagnostic performance or readiness for routine clinical use. Candidate metabolic panels still require prospective multicentre validation against established approaches, including bacteriological culture, molecular pathogen detection and somatic cell counting. Their sensitivity, specificity, predictive values and clinically meaningful decision thresholds must also be established in representative field populations.

Routine implementation would additionally require standardized procedures for milk collection, storage, spectral acquisition and data interpretation, together with evidence of analytical robustness, regulatory acceptance and cost-effectiveness under real farming conditions. Practical adoption would depend on turnaround time, compatibility with existing diagnostic workflows and acceptance by veterinarians, farmers and diagnostic laboratories. Most importantly, metabolomic testing would need to demonstrate that it provides clinically useful information beyond current methods and leads to measurable benefits, such as improved treatment selection, reduced unnecessary antimicrobial use, earlier disease detection or lower production losses. Mastitis should therefore be regarded as a promising candidate for translational evaluation, while current evidence remains insufficient to support routine clinical adoption.

#### 3.2.2. Sepsis and Systemic Infections

Sepsis is a life-threatening syndrome characterized by a dysregulated host response to infection that results in widespread tissue injury, organ dysfunction and high mortality [53]. In veterinary medicine, particularly in dogs and cats, reported mortality rates range from approximately 20% to 68%, with the poorest outcomes observed in animals presenting multiple organ dysfunction or severe physiological derangements [4]. Early recognition of sepsis is therefore essential for improving prognosis and guiding therapeutic interventions. However, diagnosis remains challenging because standardized diagnostic criteria are still lacking in veterinary medicine, and many clinical scoring systems have been adapted from human healthcare or differ considerably among studies, limiting their reproducibility and routine clinical applicability [4,54].

Sepsis is also accompanied by profound metabolic disturbances involving multiple organs and biological pathways simultaneously, making metabolomics particularly well suited to characterize its complex pathophysiology. Human metabolomics studies have consistently demonstrated alterations affecting central carbon metabolism, mitochondrial bioenergetics, amino acid utilization, lipid metabolism and oxidative stress, reflecting the extensive metabolic reprogramming associated with systemic inflammation [43,55]. Furthermore, recurrent changes in metabolites such as lactate, ketone bodies, branched-chain amino acids and lipid-derived compounds have been associated with organ dysfunction, immune activation and patient prognosis, supporting the value of metabolomic profiling for evaluating the host response during sepsis [55].

Although veterinary applications remain relatively limited, the available studies to date demonstrate the feasibility of NMR metabolomics for characterizing systemic inflammatory disorders. In neonatal calves, serum NMR metabolomics identified distinct metabolic signatures associated with sepsis, including alterations in energy metabolism, amino acid turnover and lipid-related metabolites that discriminated septic animals from healthy controls [56]. Similarly, plasma NMR metabolomics in horses revealed significant metabolic perturbations associated with systemic inflammatory response syndrome and sepsis, involving pathways related to bioenergetics, oxidative metabolism and inflammation [57].Together, these studies suggest that NMR metabolomics provides complementary information to conventional clinical assessment by capturing the global metabolic consequences of systemic inflammation.

Although veterinary evidence remains limited, the partial concordance between the available animal studies and the broader literature in human sepsis supports the translational relevance of these preliminary findings. Larger multicenter studies will now be required to validate candidate biomarkers, establish their prognostic value and determine the clinical utility of NMR metabolomics for disease stratification and therapeutic monitoring in veterinary sepsis.

#### 3.2.3. Mycobacterial Infections: Tuberculosis and Paratuberculosis

Tuberculosis (TB) and paratuberculosis (PTB) are two chronic mycobacterial diseases of major importance for animal health, animal welfare and livestock production [58,59]. In addition to their economic impact, bovine and caprine TB represent a major One Health challenge because of their zoonotic potential and the need to implement costly eradication programs based on ante-mortem diagnosis and culling of infected animals [60,61].

Despite considerable advances in diagnostic methodologies, accurate ante-mortem detection of mycobacterial infections remains challenging. TB control relies primarily on immunological assays that detect the host cellular immune response, whose performance may be influenced by multiple biological factors, including co-infections with environmental mycobacteria or Mycobacterium avium subsp. paratuberculosis (MAP), potentially reducing diagnostic sensitivity and specificity [62]. Likewise, PTB diagnosis continues to present important limitations. Although bacteriological culture remains the reference method, its routine application is hindered by the extremely slow growth of MAP, prolonged turnaround times and limited sensitivity associated with intermittent bacterial shedding [62]. These limitations have stimulated growing interest in complementary approaches capable of providing objective information on the metabolic consequences of chronic infection while improving disease characterization and biomarker discovery.

Evidence from mass spectrometry-based metabolomics and complementary omics technologies has demonstrated that chronic mycobacterial infections induce extensive biochemical remodeling involving amino acid metabolism, lipid homeostasis, energy production and immune regulation [63,64]. These alterations have been associated with granuloma formation, pathogen persistence and the modulation of host immune responses, providing a strong biological rationale for investigating the metabolic consequences of tuberculosis and paratuberculosis in naturally infected animals. Collectively, these findings indicate that chronic mycobacterial diseases generate systemic metabolic perturbations that can be detected in accessible biological matrices and are potentially exploited for disease characterization and biomarker discovery.

Consistent with this evidence, NMR metabolomics has identified disease-associated metabolic patterns in several veterinary species affected by mycobacterial infection. In cattle, serum NMR profiling discriminated against animals with TB from non-infected controls and revealed alterations involving intermediary metabolism and inflammatory pathways [65,66]. Similar findings have been reported in PTB, where NMR analysis detected perturbations in amino acid metabolism, lipid homeostasis and energy production, supporting the use of metabolic profiling as a complementary approach for disease characterization [59,65,67]. More recently, field studies in naturally infected goats demonstrated that both high-field and benchtop NMR spectroscopy can identify metabolic fingerprints associated with TB, further supporting the robustness of NMR-derived signatures across species and analytical platforms [17,65]. Although the specific advantages of benchtop NMR are discussed later in this review, these results demonstrate its potential for accessible metabolic screening under field conditions.

#### 3.2.4. Viral and Parasitic Infections

Although the application of NMR metabolomics to viral diseases in veterinary medicine remains limited, evidence from human metabolomics and experimental studies indicates that viral infections induce metabolic perturbations involving energy metabolism, amino acid utilization and lipid homeostasis [44,68]. Direct veterinary NMR studies remain limited but have characterized disease-associated metabolic alterations in naturally infected cattle with bovine respiratory syncytial virus and in dogs with canine parvoviral enteritis [69,70].

Likewise, NMR metabolomics has been applied to parasitic infections, revealing alterations in intermediary metabolism, amino acid turnover and oxidative stress that accompany chronic host–parasite interactions. For example, metabolic profiling has identified reproducible biochemical perturbations associated with protozoan infections, providing novel insights into disease pathophysiology while supporting the potential of metabolomics as a complementary tool for disease characterization [71,72].

Although the number of veterinary studies remains relatively small, the available evidence demonstrates that NMR metabolomics can be successfully applied across a broad spectrum of infectious diseases, further expanding its potential for biomarker discovery and clinical characterization in veterinary infectious medicine.

### 3.3. NMR Metabolomics in Respiratory Diseases

Respiratory diseases are among the leading causes of morbidity and economic loss in both companion animals and livestock, encompassing infectious, inflammatory and chronic pulmonary disorders. Despite major advances in diagnostic imaging and laboratory testing, the biochemical mechanisms underlying respiratory disease development and progression remain incompletely understood. In this context, metabolomics has emerged as a valuable complementary approach for investigating respiratory diseases by providing a comprehensive characterization of the metabolic alterations associated with pulmonary inflammation, tissue injury and host adaptation.

Human studies have demonstrated the potential of both NMR and mass spectrometry metabolomics for characterizing respiratory diseases including asthma, chronic obstructive pulmonary disease (COPD), acute respiratory distress syndrome (ARDS) and pulmonary infections [43,73]. Across these conditions, recurrent perturbations have been reported in pathways related to energy metabolism, amino acid utilization, oxidative stress and lipid homeostasis. Furthermore, metabolomic profiling of serum, bronchoalveolar lavage fluid and exhaled breath condensate has provided complementary insights into both systemic and local metabolic responses associated with pulmonary disease.

Veterinary applications of NMR metabolomics have so far focused primarily on inflammatory respiratory disorders. Equine asthma currently represents the best-characterized model, with NMR analysis of bronchoalveolar lavage fluid identifying reproducible metabolic alterations associated with airway inflammation and immune activation [74]. These findings demonstrate that NMR spectroscopy can characterize disease-associated metabolic changes directly within the lower respiratory tract, complementing information obtained through conventional diagnostic procedures. Similarly, plasma NMR metabolomics in experimentally infected dairy calves identified alterations in carbohydrate metabolism, amino acid turnover and inflammatory pathways following infection with two major causative agents of bovine respiratory disease [70]. Because this was an experimental infection study, the generalizability of the findings to naturally occurring, clinically heterogeneous bovine respiratory disease remains to be established.

Although veterinary evidence remains limited, the studies published to date demonstrate that NMR metabolomics can successfully characterize both local and systemic metabolic responses associated with respiratory disease. The complementary analysis of bronchoalveolar lavage fluid and blood-derived biofluids highlights the versatility of NMR metabolomics across different respiratory conditions and supports its potential for improving disease characterization as additional veterinary studies become available.

### 3.4. NMR Metabolomics in Digestive Diseases

Gastrointestinal and hepatobiliary diseases comprise a diverse group of disorders affecting both companion animals and livestock. Their multifactorial pathophysiology, together with the close interplay between host metabolism, the gut microbiota and immune regulation, makes these disorders particularly well suited for metabolomic investigation. By simultaneously capturing alterations in multiple interconnected metabolic pathways, metabolomics provides valuable insights into disease mechanisms while offering opportunities for biomarker discovery and clinical characterization.

Human metabolomics studies have demonstrated that digestive diseases, including inflammatory bowel disease, chronic liver disorders and gastrointestinal inflammation, are associated with alterations in central energy metabolism, bile acid homeostasis, amino acid utilization and metabolites derived from the gut microbiota [75,76]. Consistent with these observations, veterinary applications of NMR metabolomics have primarily focused on chronic enteropathies and hepatobiliary diseases in companion animals. In dogs with chronic inflammatory enteropathy, serum and urinary NMR metabolomics identified perturbations involving energy metabolism, amino acid metabolism and gut microbiota-derived metabolites, providing novel insights into disease-associated metabolic dysregulation [77]. Likewise, in dogs with hepatobiliary disease, NMR metabolomics revealed metabolic alterations associated with impaired liver function, dysregulated lipid metabolism and disturbances in energy homeostasis [77,78].

Although veterinary evidence remains limited, particularly in livestock species, the available studies demonstrate that NMR metabolomics can successfully characterize the metabolic consequences of chronic digestive and hepatobiliary diseases. Future studies integrating metabolomics with gut microbiome analyses will be essential to improve our understanding of host–microbiota interactions and to determine the clinical utility of NMR metabolomics for the diagnosis, prognosis and monitoring of digestive diseases across veterinary species.

### 3.5. NMR Metabolomics in Renal Diseases

Renal diseases comprise a heterogeneous group of disorders that represent an important cause of morbidity in companion animals and livestock. Among these, chronic kidney disease (CKD) is by far the most extensively investigated, particularly in aging dogs and cats, owing to its high prevalence, progressive nature and limited therapeutic options [79]. Early diagnosis and accurate monitoring of disease progression are therefore essential for delaying renal dysfunction and improving long-term clinical management. Although conventional biomarkers, including creatinine and symmetric dimethylarginine (SDMA), have substantially improved the detection of renal impairment, they provide only limited information regarding the broader metabolic disturbances associated with CKD progression [80]. Consequently, metabolomics has emerged as a valuable complementary approach for characterizing the systemic biochemical alterations associated with chronic renal disease while facilitating biomarker discovery and disease stratification.

Human metabolomics studies have shown that CKD is characterized by profound alterations in amino acid metabolism, energy homeostasis, accumulation of uremic toxins and host–gut microbiota co-metabolism [12,81]. Many of these metabolic disturbances evolve progressively with declining renal function and closely reflect the interplay between impaired kidney function, chronic inflammation and systemic metabolic dysregulation. Consistent with these observations, veterinary applications of NMR metabolomics have primarily focused on naturally occurring CKD in companion animals. In dogs, serum NMR metabolomics identified alterations involving amino acid metabolism, organic acids and metabolites associated with impaired renal function [82,83]. Similar findings have been reported in cats, where NMR metabolomics revealed metabolic signatures associated with renal dysfunction and disease severity, further supporting the applicability of this approach for CKD characterization in veterinary medicine [84].

Future longitudinal large-scale studies will be required to validate these metabolic signatures and determine whether NMR metabolomics can improve early diagnosis, disease staging and therapeutic monitoring of chronic kidney disease in veterinary practice.

### 3.6. NMR Metabolomics in Endocrine and Metabolic Diseases

Endocrine and metabolic diseases characterized by alterations in hormone production, secretion or action that ultimately disrupt systemic metabolic homeostasis [85]. In companion animals, diabetes mellitus, hyperadrenocorticism and hyperthyroidism are among the most extensively investigated endocrine disorders and are all characterized by profound alterations in carbohydrate, lipid and protein metabolism [86,87]. Because endocrine disorders directly regulate multiple interconnected metabolic pathways, they represent particularly suitable targets for metabolomic investigation.

Human metabolomics studies have extensively characterized the metabolic consequences of endocrine diseases, demonstrating alterations in glucose metabolism, lipid homeostasis, amino acid utilization and mitochondrial function [12,88]. Veterinary applications of NMR metabolomics have so far focused primarily on diabetes mellitus and hyperadrenocorticism [86,89]. In diabetic animals, NMR metabolomics identified metabolic signatures associated with impaired glucose utilization, ketone body production and dysregulated lipid metabolism [90]. More recently, NMR profiling of dogs with hyperadrenocorticism revealed alterations involving amino acid metabolism, lipid homeostasis and central energy metabolism that reflect the systemic metabolic consequences of chronic glucocorticoid excess [89].

As additional studies become available across a broader spectrum of endocrine disorders, NMR metabolomics is expected to provide valuable complementary information for understanding disease-associated metabolic alterations and improving the clinical characterization of endocrine diseases in veterinary medicine.

### 3.7. NMR Metabolomics in Musculoskeletal Diseases

Musculoskeletal diseases are among the leading causes of chronic pain, lameness and reduced athletic performance in veterinary medicine, particularly in companion animals and horses [91]. These disorders are characterized by complex interactions between inflammation, cartilage degeneration, subchondral bone remodeling and altered cellular metabolism, making them particularly suitable for metabolomic investigation [91,92]. Human studies have demonstrated that musculoskeletal diseases are associated with reproducible metabolic alterations involving inflammation, oxidative stress, energy metabolism and extracellular matrix turnover, supporting the application of metabolomics to improve disease characterization and precision medicine [93]. In veterinary medicine, these approaches have been translated primarily through the analysis of synovial fluid, which provides direct insight into the biochemical microenvironment of affected joints.

Osteoarthritis (OA) is the most prevalent musculoskeletal disease in veterinary medicine and has a major impact on animal welfare, athletic performance and economic productivity. Because conventional diagnosis is largely based on clinical examination and imaging, disease is often recognized only after irreversible structural damage has occurred. In this context, NMR metabolomics has emerged as a promising complementary approach for identifying metabolic alterations associated with early disease, disease severity and therapeutic response.

Horses currently provide the strongest evidence supporting the application of NMR metabolomics to musculoskeletal disorders. Most studies have focused on naturally occurring OA and osteochondrosis, consistently identifying alterations involving energy metabolism, amino acid utilization, lipid metabolism and oxidative stress, with recurrent changes in glucose, lactate, citrate, creatine and branched-chain amino acids [94,95]. The integration of metabolomics with proteomic and transcriptomic analyses has further improved our understanding of OA pathophysiology by linking metabolic perturbations with extracellular matrix remodeling and inflammatory pathways [95,96]. NMR metabolomics has also successfully characterized other equine musculoskeletal disorders (i.e., palmar osteochondral disease and synovitis), demonstrating the sensitivity of synovial fluid profiling for detecting disease-associated biochemical alterations [94,97].

Comparable findings have been reported on dogs. Synovial fluid NMR metabolomics distinguished osteoarthritic joints from healthy controls while identifying metabolic alterations involving energy metabolism, amino acid turnover and lipid homeostasis that were associated with disease progression [98]. Beyond OA, metabolomic profiling also identified specific intra-articular lesions, including meniscal injury secondary to cranial cruciate ligament rupture, where increased mobile lipid resonances likely reflected membrane disruption and tissue damage [99]. More recently, longitudinal metabolomic evaluation of dogs receiving undenatured type II collagen demonstrated partial normalization of the synovial metabolome following treatment, thus highlighting the potential of NMR metabolomics for monitoring therapeutic response in musculoskeletal disease [100].

Although veterinary applications remain largely focused on OA, the available evidence demonstrates that NMR metabolomics can reproducibly characterize disease-associated metabolic alterations directly within the joint environment. The accessibility of synovial fluid and the consistency of metabolic signatures reported across independent studies position musculoskeletal diseases among the most promising applications of NMR metabolomics for biomarker discovery and longitudinal assessment in veterinary medicine.

**Table 2:** Current clinical applications of NMR metabolomics in veterinary medicine.

Clinical Application	Representative Diseases	Main Species	Main Matrices	Primary Clinical Objective	Representative Direct Veterinary NMR Studies
Oncology	Mammary tumours, lymphoma, urothelial carcinoma.	Dog, cat	Urine, serum	Diagnosis, prognosis, comparative oncology	[32,33,34,37]
Infectious diseases	TB, PTB, mastitis.	Cattle, goats, dogs	Serum, milk, urine	Diagnosis, host response	[50,51,56,57,65,66,67,68,69,70,71,72]
Respiratory diseases	Equine asthma, BRD.	Horse, cattle	Serum, BALF	Diagnosis, monitoring	[70,74]
Digestive diseases	IBD, liver disease.	Dog, cat	Serum, feces	Disease characterization	[77,78]
Renal diseases	CKD, AKI.	Dog, cat	Urine, serum	Early diagnosis, monitoring	[82,83,84]
Endocrine & metabolic diseases	Diabetes, ketosis, NEB.	Dog, cat, cattle	Serum, milk	Metabolic monitoring	[87,89,90]
Musculoskeletal diseases	Osteoarthritis, laminitis	Horse, dog	Synovial fluid	Biomarker discovery	[94,95,97,98,99,100]

## 4. From Research to Clinical Implementation

**Figure 1:**
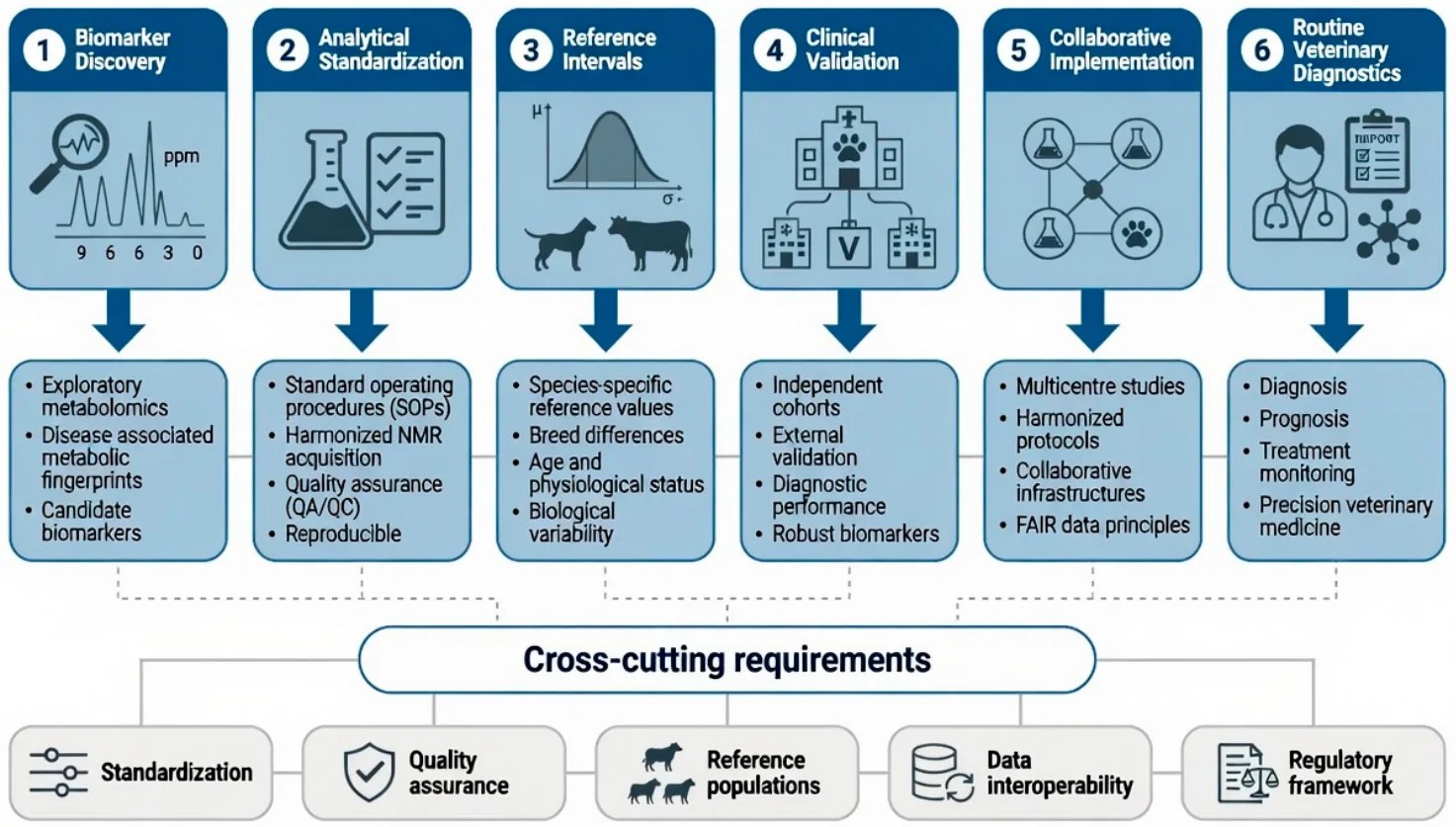
Roadmap for the clinical implementation of NMR metabolomics in veterinary medicine. The scheme summarizes six stages in the translational pathway, progressing from exploratory biomarker discovery and analytical standardization to the establishment of species-specific reference intervals, independent clinical validation, multicentre implementation and routine veterinary diagnostic use. Cross-cutting requirements include standardization, quality assurance, representative reference populations, data interoperability and appropriate regulatory frameworks.

The studies discussed in the previous sections demonstrate that NMR metabolomics can consistently identify disease-associated metabolic fingerprints across a broad spectrum of veterinary disorders. Over the past decade, the field has progressed from exploratory proof-of-concept studies to increasingly robust investigations involving larger cohorts, standardized analytical protocols and clinically relevant biological matrices. Nevertheless, despite this substantial scientific progress, the translation of NMR metabolomics into routine veterinary diagnostics remains limited. This discrepancy is not unique to veterinary medicine. Human metabolomics has followed a similar trajectory, generating thousands of candidate biomarkers while only a small fraction have reached clinical practice. These observations emphasize that successful translation depends not only on biomarker discovery but also on the establishment of analytical, biological and clinical frameworks that ensure reproducibility, robustness and clinical utility (Figure 1) [101,102].

The first prerequisite for successful clinical translation is analytical standardization. While methodological flexibility is acceptable during exploratory research, routine diagnostics require metabolomic measurements to remain comparable across laboratories, instruments and time. Consequently, every stage of the analytical workflow—from sample collection and storage to NMR acquisition, spectral processing and metabolite quantification—must be performed according to standardized operating procedures. This principle was first established through the Metabolomics Standards Initiative, which defined minimum reporting standards for metabolomic experiments, and has since been reinforced by international recommendations highlighting quality assurance, quality control and transparent reporting as essential components of reproducible metabolomics [103,104,105]. More recently, FAIR data principles have further emphasized the importance of generating metabolomic datasets that are findable, accessible, interoperable and reusable, facilitating multicenter collaboration and long-term data integration [106].

NMR spectroscopy offers several inherent advantages that facilitate this level of standardization. Compared with analytical platforms requiring extensive optimization, NMR provides excellent reproducibility, quantitative performance and high instrument-to-instrument comparability. Sample preparation is straightforward, acquisition protocols are highly standardized, and calibration procedures are well established, substantially reducing analytical variability. Recent international recommendations have further harmonized pre-analytical procedures for blood-derived biofluids, providing practical guidance for sample collection, processing and storage that facilitates reproducible metabolomic profiling across laboratories [107]. These characteristics have enabled NMR metabolomics to progress beyond exploratory research and form the basis of standardized clinical platforms in human medicine.

Analytical robustness alone, however, is insufficient for successful clinical translation. Even under fully standardized experimental conditions, metabolomic profiles remain strongly influenced by biological variability. Species, breed, age, sex, diet, physiological status and environmental exposures all contribute to shaping the normal metabolic phenotype, often generating variability comparable to—or even greater than—disease-associated metabolic changes. Distinguishing physiological variation from genuine pathological alterations therefore represents one of the principal challenges in veterinary metabolomics. A direct consequence of this biological diversity is the need for appropriate reference intervals before metabolomic biomarkers can be interpreted in a clinical setting. Unlike human medicine, where population heterogeneity is primarily influenced by age, sex and lifestyle, veterinary medicine encompasses multiple species, breeds and production systems, each characterized by distinct metabolic phenotypes. Furthermore, nutrition, reproductive status, lactation, exercise, circadian rhythms and microbiome composition all contribute to normal metabolic variation. Consequently, clinically useful metabolomic biomarkers will require species-specific and, whenever appropriate, breed-specific reference intervals established under harmonized analytical conditions [108,109].

The establishment of reference intervals also marks the transition from biomarker discovery to clinical diagnostics. Exploratory studies generally rely on statistical differences between diseased and healthy populations to demonstrate biological relevance. In contrast, routine clinical practice requires individual patient results to be interpreted against validated reference ranges that account for normal physiological variation. Developing these reference datasets therefore represents a critical step towards routine implementation and will require coordinated efforts involving standardized analytical protocols and well-characterized healthy populations. Beyond establishing appropriate reference intervals, biomarkers must also demonstrate consistent performance across independent populations. Similar challenges have been widely recognized in human metabolomics and have led to increasing emphasis on external validation, adequately powered study designs and transparent reporting before candidate biomarkers can be considered suitable for routine clinical use [102,110,111]. Fortunately, human metabolomics has already demonstrated that these objectives are achievable. Over the past decade, high-throughput NMR platforms have shown that harmonized analytical protocols, automated data processing and rigorous quality assurance enable reproducible metabolomic profiling across large populations and multiple analytical sites. The Nightingale Health platform, for example, has supported the large-scale quantification of circulating metabolites in numerous epidemiological cohorts, while the Bruker IVDr platform has established standardized workflows specifically designed for clinical laboratory environments. Together, these initiatives demonstrate that NMR metabolomics possesses the analytical robustness, scalability and reproducibility required for routine clinical applications, providing a valuable framework for the future development of veterinary NMR metabolomics [112,113].

Despite these encouraging findings, the current veterinary evidence base should be interpreted cautiously. Most published studies are exploratory, single-centre investigations involving relatively small cohorts, frequently without independent external validation. This is particularly problematic in metabolomics because the number of spectral variables or quantified metabolites may be large relative to the number of animals included. Under these conditions, feature selection, model optimization and performance assessment conducted within the same dataset can result in overfitting and information leakage, producing estimates of diagnostic accuracy that may not be reproduced in new populations. Internal cross-validation is valuable during model development but cannot replace evaluation in an independent cohort.

Translation is further complicated by the substantial biological heterogeneity of veterinary populations. Metabolic profiles may vary according to species, breed, age, sex, diet, reproductive status, production system, medication and environmental exposure. Consequently, signatures developed in one population cannot be assumed to transfer directly to another species, breed or geographical setting. Most currently reported signatures should therefore be regarded as candidate biomarkers rather than clinically validated tests. Future studies should incorporate prospective recruitment, a priori sample-size justification, representative clinical populations, rigorous control of confounding variables, nested cross-validation with feature selection performed exclusively within the training data, and validation of locked models in independent and preferably multicentre cohorts.

These achievements provide an important roadmap for veterinary medicine. Although species diversity introduces additional complexity, the fundamental principles governing clinical implementation remain unchanged. Harmonized sample collection procedures, standardized acquisition protocols, automated metabolite quantification and rigorous quality assurance can be applied across veterinary species, providing the foundation for generating comparable metabolomic data between laboratories. Their widespread adoption would facilitate multicenter collaborations, increase cohort sizes and accelerate external validation, thereby addressing several of the principal barriers that currently limit the clinical adoption of veterinary metabolomics. Beyond establishing appropriate reference intervals, biomarkers must also demonstrate consistent performance across independent populations. This process requires not only technical validation but also the definition of clinically meaningful decision limits, appropriate reference intervals, quality assurance procedures and standardized reporting formats that allow metabolomic results to be interpreted consistently by veterinarians and clinical pathologists. Although dedicated regulatory pathways for veterinary metabolomics are still evolving, existing international frameworks for bioanalytical method validation and laboratory quality management provide a solid foundation upon which future implementation strategies can be built. Ultimately, successful implementation will also depend on demonstrating that metabolomic information improves clinical decision-making beyond existing diagnostic tools.

Overall, the evidence reviewed in this review suggests that the principal barriers to the routine adoption of veterinary NMR metabolomics are no longer primarily technological. Modern NMR platforms already provide the analytical performance required for clinical applications, and numerous proof-of-concept studies have demonstrated their ability to identify robust disease-associated metabolic fingerprints across a broad range of veterinary disorders. The remaining challenges are predominantly translational and require harmonized analytical workflows, well-characterized reference populations, large multicenter validation studies and standardized clinical interpretation. Addressing these priorities will be essential to transform NMR metabolomics from a powerful research tool into a routinely applicable technology capable of supporting precision veterinary medicine.

## 5. Emerging Technologies and Collaborative Infrastructures

The successful translation of NMR metabolomics into routine veterinary practice will depend not only on standardized analytical workflows and robust clinical validation but also on continued technological innovation. In recent years, major advances in instrumentation, computational analysis and systems biology have begun to reshape the metabolomics landscape, addressing several of the practical limitations that have traditionally restricted its clinical implementation. Among these developments, benchtop NMR spectroscopy has the potential to improve accessibility and decentralize metabolomic analyses, artificial intelligence is facilitating automated interpretation of increasingly complex datasets, and multi-omics integration is providing a more comprehensive understanding of disease biology (Table 3). Rather than representing independent technological advances, these complementary approaches are expected to accelerate the transition of NMR metabolomics from specialized research laboratories to routine veterinary diagnostics.

**Table 3:** Major innovations driving the clinical implementation of veterinary NMR metabolomics.

Innovation	Barrier Addressed	Main Contribution	Remaining Challenges
Benchtop NMR	Limited accessibility	Decentralized metabolomics, simplified workflows and routine analysis	Lower spectral resolution and metabolite coverage
Artificial Intelligence	Complex data interpretation	Automated classification, prediction and clinical decision support	External validation and model interpretability
Multi-omics integration	Limited biological context	Systems-level disease characterization and precision medicine	Data integration and computational complexity
Collaborative networks (e.g., IPCN)	Lack of large validation cohorts	Standardization, multicentre studies and biomarker validation	Long-term coordination and sustainability
FAIR & federated data infrastructures	Limited data interoperability	Secure data sharing, reusable datasets and distributed analytics	Metadata harmonization and governance

### 5.1. Benchtop NMR: Bringing Metabolomics Closer to Clinical Practice

Since its introduction into biomedical research, NMR metabolomics has relied almost exclusively on high-field superconducting spectrometers, which provide excellent spectral resolution and sensitivity but require substantial financial investment, specialized infrastructure and highly trained personnel. Although these systems remain the reference standard for metabolomic research, their cost, infrastructure requirements and operational complexity have restricted their use largely to specialized research centers. Consequently, access to NMR metabolomics has traditionally been restricted to relatively few institutions, limiting its incorporation into routine veterinary diagnostics despite the growing body of evidence supporting its clinical utility.

Recent advances in permanent magnet design, radiofrequency electronics, pulse sequence optimization and spectral processing have substantially improved the analytical capabilities of benchtop NMR systems, enabling reliable metabolomic profiling of complex biofluids. Rather than replacing high-field instruments, benchtop NMR extends the analytical capabilities of metabolomics to settings where conventional superconducting systems are impractical. Lower acquisition and maintenance costs, simplified operation, minimal infrastructure requirements and improved portability have substantially reduced the barriers to adoption, opening new opportunities for implementing metabolomic analyses in veterinary hospitals, diagnostic laboratories and, potentially, field environments [17].

Importantly, the value of benchtop NMR lies not solely in increased accessibility but also in its ability to preserve many of the characteristics that make NMR particularly attractive for clinical applications. Despite operating at lower magnetic fields, modern benchtop instruments retain excellent analytical reproducibility, straightforward sample preparation and inherently quantitative measurements. While reduced spectral resolution limits the detection of low-abundance metabolites and increases signal overlap, numerous studies have demonstrated that clinically relevant metabolic fingerprints can still be identified with high diagnostic performance, particularly when combined with multivariate statistical analysis and ML approaches.

Evidence supporting the analytical feasibility of benchtop NMR has increased in both human and veterinary research. Initial studies primarily evaluated analytical performance and agreement with high-field NMR systems [63,64], whereas subsequent investigations explored its use for detecting disease-associated metabolic patterns [114,115,116]. In veterinary medicine, benchtop NMR has been applied to bovine tuberculosis, caprine tuberculosis and paratuberculosis, and feline chronic kidney disease [65,84,117]). These studies demonstrate that low-field instruments can detect metabolic differences within the populations investigated, but they do not yet establish diagnostic equivalence to high-field NMR or routine clinical utility. Most applications remain proof-of-concept studies without prospective evaluation or independent external validation.

**Figure 2:**
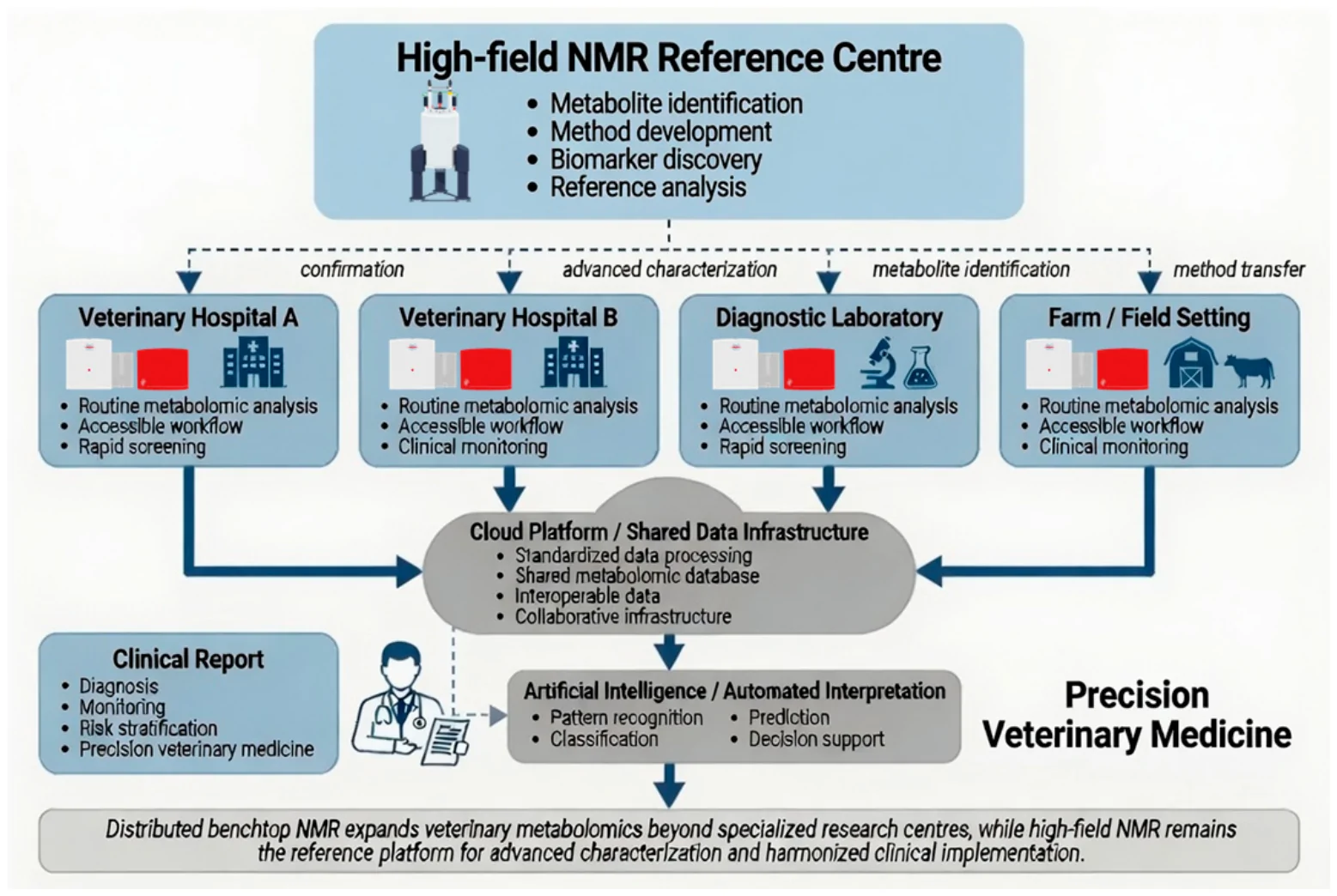
Proposed distributed model for translating benchtop NMR metabolomics into routine veterinary diagnostics. High-field NMR reference centres support metabolite identification, method development, biomarker discovery and reference analyses, whereas standardized benchtop systems located in veterinary hospitals, diagnostic laboratories and farm or field settings enable accessible routine acquisition and rapid metabolic screening. Data generated across sites are integrated through a shared infrastructure and interpreted using artificial intelligence-assisted tools to produce clinically actionable reports for diagnosis, monitoring, risk stratification and precision veterinary medicine.

Beyond these demonstrated proof-of-concept applications, benchtop NMR could potentially extend metabolic profiling to veterinary hospitals, diagnostic laboratories and livestock settings where superconducting high-field instruments are impractical. Its lower infrastructure requirements, comparatively simple operation and compatibility with automated sample handling may facilitate multicentre research and higher-throughput analyses. Potential future applications include near-patient testing, field-based disease surveillance, herd-health monitoring and precision farming, as summarized in Figure 2. These possibilities, however, remain largely prospective. Although high-field NMR will remain essential for metabolite identification, method development and comprehensive metabolic characterization, the role of benchtop NMR in routine veterinary diagnosis remains to be established through prospective multicentre studies, external validation of locked diagnostic models, comparison with existing tests, standardized quality-assurance procedures and formal assessment of clinical utility and cost-effectiveness.

### 5.2. Artificial Intelligence for Automated Metabolomic Interpretation

The increasing complexity of metabolomic datasets has transformed data interpretation into one of the principal challenges limiting the routine clinical implementation of NMR metabolomics. Modern metabolomic studies routinely quantify dozens or hundreds of metabolites simultaneously, generating multidimensional datasets that frequently exceed the interpretative capacity of conventional statistical approaches. In this context, artificial intelligence (AI) and machine learning (ML) have emerged as powerful tools for extracting clinically relevant information from complex metabolic profiles, facilitating disease classification, patient stratification and outcome prediction [118,119].

Rather than focusing on individual metabolites as isolated biomarkers, AI-based approaches analyse the entire metabolic fingerprint, identifying subtle multivariate patterns that may remain undetected using traditional statistical methods. This system-level interpretation is particularly well suited to metabolomics, where disease-associated alterations generally involve coordinated changes across multiple interconnected metabolic pathways rather than large variations in single compounds. Consequently, ML algorithms are increasingly being incorporated into metabolomic workflows to improve diagnostic accuracy, estimate disease prognosis and identify clinically meaningful metabolic phenotypes.

Beyond improving predictive performance, AI is also transforming the practical implementation of metabolomics. Automated data processing, spectral deconvolution, metabolite annotation and quality assessment reduce operator dependency while increasing analytical reproducibility and throughput. More recently, advances in explainable AI have begun to address one of the principal limitations of complex ML models by providing transparent interpretations of metabolic features contributing to model predictions. This represents an important step towards clinical adoption, where interpretability and confidence in automated decision-support systems are essential for acceptance by clinicians and laboratory specialists [120,121].

However, predictive performance may be overestimated when high-dimensional metabolomic data are analysed in relatively small cohorts. Overfitting occurs when a model captures study-specific variation rather than generalizable biological information, whereas data leakage may arise when preprocessing, feature selection or hyperparameter optimization uses information from validation or test samples. To minimize these risks, normalization, scaling, imputation and feature selection should be performed separately within each training fold. Nested cross-validation can separate model optimization from performance estimation, while permutation testing can help determine whether the observed discrimination exceeds that expected by chance. Ultimately, generalizability should be evaluated by applying a locked model, without further modification, to an independent external cohort. Feature-importance and explainability methods can facilitate interpretation of model predictions, but they do not substitute for external validation or establish biological causality.

Although most applications have so far been developed in human medicine, initial veterinary studies have explored the potential of AI-assisted metabolomics. In a cohort of 139 client-owned dogs, the integration of NMR-derived metabolite and lipoprotein measurements with haematological and clinical variables enabled discrimination between healthy and diseased animals using repeated internal cross-validation [37]. These findings demonstrate feasibility but require confirmation using a locked model in an independent external cohort before their clinical utility can be established.

The role of AI should therefore be viewed as complementary rather than competitive with NMR metabolomics. While NMR provides robust, quantitative and highly reproducible metabolic measurements, AI facilitates the interpretation of increasingly complex datasets and their translation into clinically actionable information. As larger standardized metabolomic databases become available, the integration of automated interpretation with harmonized analytical workflows is expected to play a central role in the implementation of precision veterinary medicine.

### 5.3. Multi-Omics Integration

Although metabolomics provides the closest molecular representation of the phenotype, disease development results from complex interactions occurring across multiple biological layers, including the genome, transcriptome, proteome and microbiome. Consequently, no single omics technology can fully capture the biological complexity underlying health and disease. Integrating NMR metabolomics with complementary molecular datasets has therefore emerged as a key strategy for improving disease characterization, identifying molecular mechanisms and supporting precision veterinary medicine [122,123].

Within this multi-omics framework, NMR metabolomics occupies a particularly valuable position because it reflects the downstream consequences of genetic, transcriptional and proteomic alterations while simultaneously capturing the influence of environmental factors such as nutrition, management practices and host–microbiome interactions. Rather than competing with other analytical platforms, metabolomics complements them by providing functional information that links molecular regulation with the observable phenotype. This integrative approach is particularly attractive in veterinary medicine, where disease expression is influenced not only by genetics but also by species, breed, production systems and environmental conditions.

Among the different integration strategies, the combination of NMR and mass spectrometry has attracted particular attention. While mass spectrometry offers superior sensitivity and broader metabolite coverage, NMR provides highly reproducible, inherently quantitative measurements with minimal sample preparation. Their complementary analytical characteristics improve metabolite identification, increase metabolome coverage and strengthen biological interpretation, making integrated metabolomic workflows increasingly attractive for both research and clinical applications [124].

Looking forward, the integration of metabolomic profiles with genomic information, microbiome composition, imaging biomarkers and clinical records is expected to provide a more comprehensive characterization of disease than any individual modality alone. As standardized analytical workflows, interoperable databases and artificial intelligence continue to evolve, multi-omics integration will play an increasingly important role in biomarker discovery, patient stratification and the implementation of precision veterinary medicine.

### 5.4. Collaborative Networks and Data Infrastructures

The successful clinical implementation of veterinary NMR metabolomics will depend not only on technological innovation but also on the establishment of collaborative research infrastructures capable of generating large, standardized and interoperable datasets. Although numerous studies have demonstrated the diagnostic and prognostic potential of NMR metabolomics across a broad range of veterinary diseases, most remain limited by relatively small cohort sizes, single-center recruitment and restricted external validation. Moving beyond proof-of-concept studies will therefore require coordinated international networks operating under harmonized analytical protocols, standardized quality assurance procedures and common data standards.

Human metabolomics has already demonstrated the feasibility of this collaborative approach through the development of dedicated metabolic phenotyping centers and their subsequent integration into international research infrastructures. The National Phenome Centre, established at Imperial College London, pioneered the concept of large-scale metabolic phenotyping based on standardized analytical workflows, centralized quality management and common operating procedures. Its subsequent evolution into the International Phenome Centre Network (IPCN) extended these principles across geographically distributed laboratories, creating a collaborative infrastructure capable of generating directly comparable metabolomic data through harmonized analytical methodologies and shared governance. Rather than functioning simply as a consortium of collaborating laboratories, the IPCN illustrates how permanent research infrastructures can support continuous biomarker discovery, validation and clinical translation through long-term harmonization of analytical practices [125].

A cornerstone of this strategy is the adoption of standardized analytical platforms that ensure reproducibility across participating centers. Harmonized sample preparation, unified acquisition protocols, rigorous quality assurance procedures and standardized spectral processing enable metabolomic data generated at different laboratories to be directly comparable. One of the best examples of this philosophy is the implementation of the Bruker IVDr framework, which provides standardized NMR workflows specifically designed for large-scale clinical metabolomics and has become a key analytical platform within the IPCN. More recently, this collaborative model has begun to expand towards standardized benchtop NMR workflows, reflecting the growing interest in extending harmonized metabolomic analyses beyond specialized phenome centers into routine clinical laboratory environments.

The long-term success of collaborative metabolomics will also depend on digital infrastructures that facilitate data interoperability and large-scale analysis. Public repositories such as MetaboLights, together with the adoption of FAIR (Findable, Accessible, Interoperable and Reusable) data principles and standardized metadata, provide the foundations for integrating metabolomic datasets generated across independent studies while maximizing data reuse, reproducibility and transparency [106,126,127]. Federated data infrastructures further extend this concept by allowing predictive models to be developed across geographically distributed datasets without requiring centralized transfer of sensitive information, thereby facilitating international collaboration while preserving local data governance.

These collaborative infrastructures are likely to be particularly valuable in veterinary medicine, where biological diversity across species, breeds, production systems and geographical regions makes large, heterogeneous datasets essential for robust biomarker validation. International networks built upon harmonized analytical protocols, interoperable databases and standardized quality frameworks will facilitate the establishment of reference metabolomic libraries, the external validation of diagnostic models and the development of clinically reliable decision-support systems. The success of large-scale initiatives such as the UK Biobank metabolomics programme illustrates the scientific value of harmonized population-scale metabolomic resources for biomarker validation and clinical translation, providing a framework that could inspire future international veterinary metabolomics networks [112].

## 6. Conclusions

NMR metabolomics has undergone substantial development over the past two decades and has become a robust analytical platform for veterinary research. The evidence reviewed in this article shows that NMR can detect disease-associated metabolic phenotypes across a range of disorders in companion and production animals. However, for most clinical applications, evidence supporting diagnosis, prognosis or therapeutic monitoring remains exploratory and has not yet been confirmed in independent, clinically representative cohorts.

Further biomarker discovery remains necessary in several areas of veterinary medicine. At the same time, a central challenge is determining which reported metabolites and multivariate signatures are analytically robust, biologically reproducible and clinically useful. Addressing this challenge will require harmonized analytical methodologies, appropriate quality-assurance frameworks, prospective multicentre studies and external validation of predefined and locked diagnostic models.

Advances in benchtop instrumentation, computational analysis, multi-omics integration and collaborative research infrastructures provide important opportunities to address some of the limitations that have historically restricted NMR metabolomics to specialized research laboratories. Nevertheless, the contribution of these developments to routine veterinary practice must be established through comparison with existing diagnostic approaches, definition of clinically relevant decision thresholds, evaluation of cost-effectiveness and demonstration that metabolomic information improves clinical decision-making.

The future contribution of veterinary NMR metabolomics will therefore depend on the ability of the scientific community to establish common analytical standards, share well-characterized and interoperable datasets, and evaluate candidate biomarkers in appropriately designed clinical studies. If these objectives are achieved, NMR metabolomics could become a useful component of precision veterinary medicine, contributing to disease characterization, animal-health management and comparative research within the One Health framework.

## Figures and Tables

**Table 1:** Comparative characteristics of NMR- and mass spectrometry-based metabolomics. The relative performance of each platform depends on the instrument, analytical configuration, biological matrix and intended clinical application. NMR and MS should therefore be regarded as complementary rather than competing technologies.

Criterion	NMR-Based Metabolomics	MS-Based Metabolomics	Translational Implication
Analytical sensitivity	Lower sensitivity; primarily detects metabolites present at relatively high concentrations	Substantially higher sensitivity, enabling detection of low-abundance and trace metabolites	MS provides deeper discovery coverage; NMR captures a smaller but highly reproducible metabolic profile
Metabolite coverage	Moderate and relatively consistent; signal overlap may limit resolution	Broad, potentially covering hundreds or thousands of features depending on chromatography and ionization	MS is preferable for comprehensive discovery, whereas NMR supports standardized profiling
Reproducibility	High analytical and interlaboratory reproducibility under standardized protocols	High for validated targeted assays, but untargeted workflows are more affected by chromatography, ion suppression and batch effects	NMR is particularly suitable for longitudinal and multicentre studies
Quantification	Inherently quantitative with an appropriate reference and a broad linear dynamic range	Absolute quantification generally requires calibration curves and isotope-labelled internal standards; untargeted measurements are often relative	NMR facilitates standardized absolute measurements; targeted MS can provide highly sensitive quantification
Cost and infrastructure	High initial cost for superconducting systems but relatively low consumable costs; benchtop systems reduce infrastructure requirements	Instrument costs vary widely, with recurring expenditure for solvents, columns, gases, standards and source maintenance	The most economical option depends on throughput, analytical scope and whether targeted or untargeted analysis is required
Clinical applicability	Strong potential for automated, high-throughput and harmonized profiling, but limited by lower sensitivity	Already established for many targeted clinical assays; untargeted clinical standardization remains more demanding	The platforms are complementary and may be combined according to the intended clinical use

## Data Availability

No new data were created or analyzed in this study.

## Abbreviations

The following abbreviations are used in this manuscript:

GC–MS	Gas Chromatography–Mass Spectrometry
IPCN	International Phenome Centre Network
MAP	Mycobacterium avium subsp. paratuberculosis
ML	Machine Learning
NMR	Nuclear Magnetic Resonance
OA	Osteoarthritis
PTB	Paratuberculosis
SDMA	Symmetric Dimethylarginine
TB	Tuberculosis
